# The Lack of SNARE Protein Homolog Syn8 Influences Biofilm Formation of *Candida glabrata*

**DOI:** 10.3389/fcell.2021.607188

**Published:** 2021-02-12

**Authors:** Xinyue Chen, Shun Iwatani, Toshitaka Kitamoto, Hiroji Chibana, Susumu Kajiwara

**Affiliations:** ^1^School of Life Sciences and Technology, Tokyo Institute of Technology, Yokohama, Japan; ^2^School of Materials and Chemical Technology, Tokyo Institute of Technology, Yokohama, Japan; ^3^Medical Mycology Research Center, Chiba University, Chiba, Japan

**Keywords:** *Candida glabrata*, biofilm formation, genetic screening, SNARE protein, vacuole

## Abstract

Biofilm formation of *Candida* species is considered to be a pathogenic factor of host infection. Since biofilm formation of *Candida glabrata* has not been as well studied as that of *Candida albicans*, we performed genetic screening of *C. glabrata*, and three candidate genes associated with biofilm formation were identified. *Candida glabrata SYN8* (CAGL0H06325g) was selected as the most induced gene in biofilm cells for further research. Our results indicated that the *syn8*Δ mutant was defective not only in biofilm metabolic activity but also in biofilm morphological structure and biomass. Deletion of *SYN8* seemed to have no effect on extracellular matrix production, but it led to a notable decrease in adhesion ability during biofilm formation, which may be linked to the repression of two adhesin genes, *EPA10* and *EPA22*. Furthermore, hypersensitivity to hygromycin B and various ions in addition to the abnormal vacuolar morphology in the *syn8*Δ mutant suggested that active vacuolar function is required for biofilm formation of *C. glabrata*. These findings enhance our understanding of biofilm formation in this fungus and provide information for the development of future clinical treatments.

## Introduction

*Candida glabrata*, identified as a non-*albicans Candida* (NAC) species, is one of the common causes of systemic candidiasis and shows resistance to most azole drugs (Rodrigues et al., 2014; Pristov and Ghannoum, 2019). However, studies on the mechanisms of virulence in *C. glabrata* are far fewer than those in *C. albicans*. The biofilm of *Candida* species is known to be a pathogenic factor in human infections and difficult to eradicate in healthcare settings (Douglas, 2003; Eix and Nett, 2020). In *C. glabrata*, the biofilm is composed of only yeast-shaped cell clusters packed in a multilayer, which is quite different from biofilms of *C. albicans* with their elongated filamentous forms (Brunke and Hube, 2013). Because *C. glabrata* lacks filamentous growth, it is important to investigate its own distinctive biofilm pathogenicity.

Biofilm formation is a complex process that includes adhesion, colonization, extracellular matrix production, biofilm maturation, and dispersal (Ramage et al., 2009). The first step is *Candida* cell adhesion to the surfaces of host cells and biomaterials such as silicone. Therefore, many previous studies have investigated the roles of adhesins at the cell surface in biofilm formation. The *EPA* (epithelial adhesin) gene family is a major group of adhesins in *C. glabrata.* Because the *C. glabrata* haploid genome is more closely related to *Saccharomyces cerevisiae* than *C. albicans*, this large *EPA* family is similar to the *FLO* gene family of *S. cerevisiae*, which encodes lectins for flocculation (de Groot et al., 2008). In particular, *EPA3* and *EPA6* have been indicated as encoding the main adhesins involved in biofilm formation of *C. glabrata* (Iraqui et al., 2005; Cavalheiro et al., 2018). Furthermore, *EPA6* expression is positively regulated by a Yak1p kinase that acts through a subtelomeric silencing pathway and by the chromatin remodeling Swi/Snf complex, whereas it is negatively regulated by the transcription factor Cst6p (Iraqui et al., 2005; Riera et al., 2012). The Swi/Snf nucleosome-remodeling complex interacts with the nucleosomes via the Snf2p and Snf6p components. *SNF2* and *SNF6* gene disruption mutants also exhibit poor ability to form biofilms (Riera et al., 2012). Pgs1p is a phosphatidylglycerol phosphate synthase, and it has been reported that deletion of the *PGS1* gene results in increased cell surface hydrophobicity and decreased biofilm formation of *C. glabrata* (Bat’ová et al., 2009). In addition to proteins that act on cell wall synthesis related to the adhesion step during biofilm formation, some recent studies have reported that the multidrug resistance transporters Pdr16p and Tpo1_2p contribute to azole resistance and are related to biofilm formation (Culakova et al., 2013; Santos et al., 2017). More recently, Dtr1p, Qdr2p, and Tpo4p drug:H + antiporters were found to be associated with biofilm formation of *C. glabrata* (Widiasih Widiyanto et al., 2019; Santos et al., 2020). Thus, it seems like a broad range of proteins, which are valuable to study, affect the process of biofilm formation of *C. glabrata*.

Comprehensive screening of biofilm-related genes in fungi was commonly used the collections of gene mutants (Iraqui et al., 2005; Vandenbosch et al., 2013), and the genetic expression analysis comparing the biofilm form with planktonic cells (García-Sánchez et al., 2004; Nobile et al., 2012). However, these previous studies have mostly used *C. albicans* and *S. cerevisiae* rather than *C. glabrata.* The aim of this study was to identify new genes involved in biofilm formation of *C. glabrata.* Through a comprehensive screening of 101 *C. glabrata* mutants, three gene mutants exhibited less than 70% of the metabolic activity compared with the wild type strain. Our study focused on one of the new genes, *SYN8* (CAGL0H06325g), which encodes a SNARE (soluble N-ethylmaleimide-sensitive factor attachment protein receptor) protein in *C. glabrata*. Although there have been many studies on the roles of cell wall proteins in the biofilm formation of *C. glabrata*, the relationship between biofilm formation and SNARE proteins is unclear. This is the first study to investigate the role of the SNARE Syn8p in *C. glabrata*, and we demonstrated that this gene is involved in normal vacuolar function. In addition, our results suggested that *SYN8* influences the biofilm formation of *C. glabrata*.

## Materials and Methods

### Strains and Media

The *C. glabrata* strains used in this study are listed in Table 1 and Supplementary Table 1. All yeast strains were typically grown in YPD medium (1% Bacto yeast extract, 2% Bacto-peptone and 2% glucose) or CSM medium (pH 5.8) (0.67% Bacto Yeast Nitrogen base without amino acids, 0.079% CSM complete supplement mixture, and 2% glucose) at 37°C. Solid media were supplemented with 2% agar. Chemicals used in these experiments were obtained from Nacalai Tesque, Difco Laboratories, and Wako.

**TABLE 1:** Strain list.

Strain	Parent	Genotype	References
CBS138		Wild type strain	Ueno et al., 2007, 2011
2000H	2001U	*his3:ScURA3 ura3*	Ueno et al., 2007, 2011
KUE100	2000H	*his3 yku80:SAT1 flipper*	Ueno et al., 2007, 2011
*syn8*Δ	KUE100	*his3 yku80:SAT1 syn8:CgHIS3*	This study
*SYN8*	*syn8*Δ	*his3 yku80:SAT1 SYN8:CgHIS3*	This study

### Plasmid Construction

**FIGURE 1:**
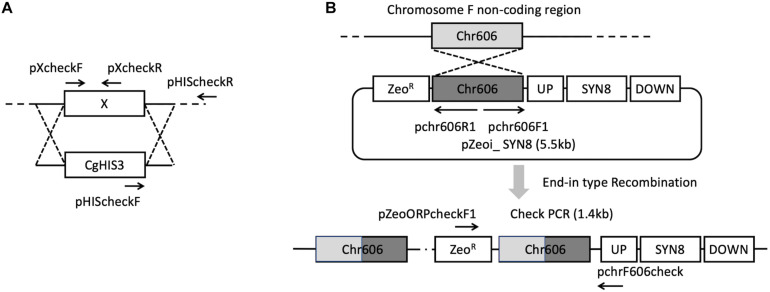
Strategies for gene deletion **(A)** and *SYN8* reintegration **(B)**.

The *C. glabrata* mutant library was constructed using the method described previously (Ueno et al., 2007), and the information of gene sequence was provided by Candida Genome Database (Koszul et al., 2003). The gene deletion was carried out in the parental strain KUE100. The target gene was replaced by a DNA replacement cassette including the *CgHIS3* gene through homologous recombination. The pHIS906 plasmid including *CgHIS3* was used as a template (Figure 1A). Recombination loci and gene deletion were verified by PCR.

To construct the *SYN8* reintegrated strain from the *syn8*Δ mutant, *C. glabrata* wild type genomic DNA was extracted from the CBS138 strain and used as the template to amplify the *SYN8* ORF with a 500 bp promoter and 200 bp terminator. The primers pSyn8compF and pSyn8compR were designed to amplify the *SYN8* (1.5 kb) fragments. Next, the plasmid pZeoi_comp606 and the amplified 1.5 kb fragment were digested, respectively, and then ligated together to construct pZeoi_SYN8 (Figure 1B). The sequence of the *SYN8* gene was confirmed by a DNA sequencer (Open Research Facilities for Life Science and Technology, Tokyo Institute of Technology). The primers pChr606F1 and pChr606R1 were used to amplify the cassette from pZeoi_SYN8, and the purified DNA product was transformed into the non-coding region on chromosome F position 605,901–606,015 of the mutant *syn8*Δ. Successful integration at the right position on the transformant genome was confirmed by the primers pZeoORFcheckF1 and pchr606check. All primer sequences used in this experiment are shown in Table 2 and designed by the software Primer 3.

**TABLE 2:** Primers for *SYN8* deletion and reintegration.

Primers	Primer sequence (5′→ 3′)
pSyn8F	TTGCGTTTGAACCCCGGGATTAAGGATTGGATTACTGTAT ACAAGCAGCGAAGGAGGGCCGCTGATCACG
pSyn8R	GAAATATTGAATGGTAGTATCTGTAATAATGGTTAATCTCAT CAAACTGAGATGCTCATCGTGAGGCTGG
pHIScheckF	CAGCCATTGTATGCCACTGTATGA
pHIScheckR	CAGCTTTATCTCAGAAAACCAG
pSyn8checkF	GGATCTGAAGGTTTCATATGAGCTTGA
pSyn8checkR	GAAAAGTCATTGGGGCTGATGCA
pSyn8compF	AATCTAGAATTCTATCCTCCATGGGCTCCTT
pSyn8compR	AATCTAGATCTGTGATGAGCCGAATCAGAATC
pChr606 F1	AAGAATGCCAACCAAGGATTCACAATAATCCGAAGC
pChr606 R1	TTAGGCAAAGCATTTGTAAACCATTACAAGCACTC
pZeoORFcheckF1	AAGTTGACCAGTGCCGTTCCGGTG
pchr606check	CTAATGGGGATATAGAAAGATAGGG

### Transformation of *C. glabrata*

After constructing the integration cassettes, the cassettes were introduced into the *C. glabrata syn8*Δ strain based on a previous method with slight modification (Ueno et al., 2011). The strain colonies were incubated in YPD liquid medium and cultured overnight with shaking at 37°C. The cultured cells were resuspended in 10 mL fresh YPD medium and grown from an OD_600_ of 0.4–1.0 at 37°C. These cells were resuspended in 10 mL of 0.15 M lithium acetate dissolved in TE buffer (LiOAc/TE) and shaken lightly for 1 h at 37°C. Then the cells were again harvested and resuspended in 400 μL of 0.15 M LiOAc. Sixty microliters of the cell suspension were supplemented with 20 μg single-stranded carrier DNA and 5–10 μg integration cassette DNA solution and mixed gently in a new 1.5 mL tube. The cell suspension was incubated at 37°C with rotation for 37 min and then mixed completely with 120 μL of 52.5% polyethylene glycol 4000, 0.15 M LiOAc by pipetting. The cells were incubated again for 45 min at 37°C and heat-shocked at 42°C for 15 min. Then, the cells were harvested and incubated in 1 mL of fresh YPD liquid medium for 12 or 24 h at 37°C with shaking for recovery. After that, cells were spread on YPD plates containing zeocin (50–200 μg/mL) and incubated at 37°C for 1–2 days.

### Biofilm Formation and Plate Siliconization

*C. glabrata* biofilms were formed according to an *in vitro* method using a 96-well flat-bottom polystyrene plate (IWAKI, Japan) (Widiasih Widiyanto et al., 2019). Yeast cells were cultured overnight at 37°C. Cells were harvested by centrifugation and washed twice in 100 μL sterile phosphate-buffered saline buffer (PBS, 137 mmol/L NaCl, 2.7 mmol/L KCl, 10 mmol/L Na_2_HPO_4_, 2 mmol/L KH_2_PO_4_, pH 7.2). Then cells were resuspended in fresh medium and standardized to a concentration of 10^7^ cells/mL. Each well of the plate was filled with a 100 μL aliquot of cell suspension, and the plates were incubated at 37°C for 1.5 h to allow the cells to adhere to the surfaces of the wells. Following the adhesion phase, the cell suspensions were removed, and the wells were washed with PBS buffer to remove non-adherent cells. Fresh medium (100 μL) was added to each well, and then the plate was incubated at 37°C for 24 h. The siliconized plates were also used to compare the influence of the silicone material on biofilm formation. Three hundred microliters of Sigmacote (Sigma-Aldrich, United States) were added into the well of a plate and distributed over the well according to the product information. Because the reaction was almost instantaneous, the rest of the Sigmacote was quickly removed. After all of the wells of one plate were treated, the whole plate was dried in a hood with UV sterilization. The silicone is covalently bound to the well.

### XTT Reduction Assay

2,3-Bis(2-methoxy-4-nitro-5-sulfophenyl)-5-[(phenylamino) carbonyl]-2H-tetrazolium-5-carboxanilide (XTT) reduction assay was used to measure the metabolic activity of biofilms of *C. glabrata* as described previously (Chen et al., 2017). Briefly, after washing the biofilm with 100 μL PBS buffer, a 100 μL aliquot of XTT-menadione was added into each well. The plate was then incubated for 2 h at 37°C, and the colorimetric change was measured at 490 nm by a microtiter plate reader (VarioskanLUX, ThermoFisher, Japan). Negative controls were the unseeded wells. The final result for each strain was confirmed at least three independent times.

### Observation of Biofilms by SEM

To observe the microstructures of *C. glabrata* biofilms, sterile 3-mm cubic silicone sponges (AS ONE Corp., Japan) were prepared and put into a 96-well plate before cell cultivation. Aliquots (200 μL) of precultured cell suspensions were added in the plate, and the biofilm was allowed to form subsequently on the silicone sponges. These samples were treated as described before (Chen et al., 2017). The biofilm microstructures of *C. glabrata* were observed by a desktop scanning electron microscope, Phenom^TM^ -Pro-X (Phenom-World, Netherlands).

### Biofilm Dry Weight and Matrix Analysis

Biofilms for matrix collection were formed in 12-well polystyrene microtiter plates (IWAKI, Japan). Three-milliliter aliquots of cell suspensions that were diluted to 1 × 10^7^ cells/mL were filled in each well. After incubation at 37°C for 24 h, the matrix of each biofilm was collected using a previously described method with slight modification (Silva et al., 2009; Taff et al., 2012). Biofilms were scraped from the wells and resuspended with 5 mL distilled water in tubes. The tubes were sonicated for 10 min, vortexed vigorously for 5 min, and centrifuged at 5,000 × *g* for 10 min at 4°C. The supernatant was transferred into new tubes to separate cell pellets from soluble matrix material and stored at −20°C. To measure the biofilm dry weight, the remaining pellets were dried at 60°C until a constant weight. The supernatant samples were used to determine the contents of proteins and total carbohydrates. The protein content of each biofilm matrix was measured by using Protein Assay CBB Solution (5× ) (Nacalai Tesque, Japan). Bovine serum albumin (BSA) (2.5–25 μg/mL) was used to make the standard curve for the protein test. The absorbance was read at 595 nm. The total carbohydrate content of each biofilm matrix was detected based on the phenol-sulfuric acid method (Dubois et al., 1956). Briefly, 1 mL sample was mixed well with 250 μL 80% phenol (wt/wt in H_2_O) and added rapidly with 2.5 mL H_2_SO_4_. Finally, after the tubes cooled down to room temperature, the samples were mixed well and the absorbance was read at 490 nm. Glucose (5–500 μg/mL) was used to make the standard curve for the total carbohydrate test.

### Real-Time PCR

The planktonic cells were cultured in CSM medium at 37°C. The culture was diluted to an OD_600_ of 0.2 in 50 mL fresh CSM medium before being grown to OD_600_ of 1∼2. The biofilm cells were induced to form in 12-well plates for 24 h as described above. Total RNA was extracted from unicellular cells and biofilm cells using the glass bead lysis method. These RNAs were used as templates to synthesize first strand cDNA using the ReverTra Ace qPCR RT Master Mix with gDNA Remover kit (TOYOBO, Japan). Real-time PCR was performed by using the THUNDERBIRD SYBR qPCR Mix kit (TOYOBO, Japan) and gene expression levels were determined by using ΔΔC_t_ method. The gene expression values were normalized by comparison to the expression of a housekeeping gene, *CgACT1* (Pfaffl, 2001). All the primers for real-time PCR reactions were listed in Table 3.

**TABLE 3:** Primers for RT-qPCR.

Primers	Primer sequence (5′→ 3′)
ACT1F	CCTACGAATTGCCAGATGGT
ACT1R	ACAGATGGGTGGAACAAAGC
SYN8F	CATCAGCCCCAATGACTTTT
SYN8R	TGCCAAGGAATCAAGATGTG
EPA1F	GATTGCTGCAGAAGGGATTC
EPA1R	CTGTTTTTGAGCCCCAGATG
EPA6F	ATCAGGATCGAATCCATGTTG
EPA6R	ACAGCGAAGTACACCCCATT
EPA10F	GGGACAGACCACGATCACTT
EPA10R	AACCACCACCAGGAACCATA
EPA22F	ACTTGGCGAGTACGTTGCTT
EPA22R	TGATCCGGAACGGAATAGAG

### Susceptibility to Drugs and Ions

Cells were cultured in 5 mL YPD medium overnight at 37°C. The overnight cultures were then diluted to an OD_600_ of 0.1, which was used as a starting point for 10-fold serial dilutions. Three microliters of each serial dilution were spotted onto YPD plates containing drugs and ions (Banuelos et al., 2010; Palanisamy et al., 2010). The final concentrations of each drug were 0.01% SDS, 70 μg/mL hygromycin B, 80 μg/mL calcofluor white, and 100 μg/mL Congo red. Concentrations of ions were as follows: 4 mM ZnCl_2_, 10 mM MnCl_2_, 1 M NaCl, 1 M KCl. The plates were then incubated at 37°C for 24–60 h. Chemicals used in these experiments were obtained from Nacalai Tesque, Difco Laboratories, Wako, and Funakoshi in Japan.

### Antifungal Susceptibility of Unicellular Cells by Broth Dilution

Minimum inhibitory concentration (MIC) assays were prepared based on EUCAST guidelines (Rodríguez-Tudela et al., 2003). Briefly, cells were cultured overnight in CSM medium, washed twice by PBS buffer, and resuspended in RPMI medium to a concentration of 10^4^ cells/mL. Volumes of 100 μL of cells including hygromycin B were filled in 96-well microtiter plate wells. The concentration range of the 2-fold drug dilutions was from 7.8125 to 500 μg/mL. The plates were incubated at 37°C for 24 h and then read by a microtiter plate reader at 530 nm.

### Antifungal Susceptibility of Biofilm Cells by XTT Assay

Sessile Minimum Inhibitory Concentration (SMIC) of biofilm cells was tested as previously published (Ramage et al., 2001). Biofilms were formed as described above. After 24 h, the suspension was discarded, and wells were washed twice by 100 μL PBS buffer. In order to avoid disruptions of biofilms, different concentrations of 2-fold antifungal agent dilutions were prepared separately in medium (62.5–1,000 μg/mL), and 100 μL amounts of each antifungal concentration were added into the wells. Controls were prepared with biofilm wells that were untreated with drugs or unseeded wells with medium only. The plates were incubated at 37°C for 24 h again. After 24 h, the plates were carefully washed with 100 μL PBS. To determine XTT activity, 100 μL of a 1 mg/mL solution of XTT-menadione was put into each well and the plates incubated for 2 h. Absorbance at 492 nm was measured by a microtiter plate reader. The lowest concentrations associated with a 50 or 90% reduction in absorption compared with the untreated biofilm wells were reported as the MIC_50_ or MIC_90_.

### Visualization of Vacuolar Morphology

To visualize the cell vacuolar morphology, the fluorescent dye FM4-64 [N-(3-triethylammoniumpropyl)-4-(6-(4-(diethylamino) phenyl) hexatrienyl) pyridinium dibromide] (SynaptoRed C2) (Wako, Japan) was used to stain vacuolar membranes (Vida and Emr, 1995). The cells were cultured for 16 h overnight and inoculated into fresh medium for more 6 h until log phase. Then, cells were resuspended to 2–4 OD_600_ in fresh medium with 40 μM FM4-64 and incubated for 15 min at 37°C. After this preliminary labeling step, the cells were harvested at room temperature and resuspended to 1 OD_600_ in fresh medium and incubated for 45 min at 37°C. After this, the cells were resuspended to 2–4 OD_600_ and observed by confocal laser scanning microscopy (CLSM) LSM780 (ZEISS, Japan). Red fluorescence filters (excitation filter, 533–588 nm; barrier, 608–683 nm) were used to visualize vacuolar membranes stained with FM4-64.

## Results

A comprehensive screening of *C. glabrata* mutants was performed by using our *C. glabrata* mutant library. The null mutants of 101 genes that encode proteins including signal peptides or transmembrane domains were selected and screened for their biofilm formation abilities. The biofilm formation abilities of 101 mutants were compared with that of the reference strain (Supplementary Figure 1 and Supplementary Table 1). In comparison with the wild type, three gene mutants of *C. glabrata* showed significant reductions in biofilm formation, and the metabolic activities of these mutant biofilms were less than 70% of that of the wild type strain. The expressions of all three of these genes in *C. glabrata* during biofilm formation were detected as upregulated compared with those in planktonic cell conditions (Supplementary Figure 2). In particular, the expression levels of two genes in biofilm formation were more than two times higher than in planktonic cells. From a search of the *Candida* genome database, we found that one gene is a homolog of the SNARE protein family of *S. cerevisiae.* Since this gene showed the most drastic induction in the biofilm cells, we focused on this gene *SYN8* (CAGL0H06325g) and characterized its deletion as the *syn8*Δ mutant. *SYN8* in *C. glabrata* is a homolog of *SYN8* in *S. cerevisiae* and of CR_03140C_A/*SYN8* in *C. albicans*. In this study, the protein sequence of *C. glabrata SYN8* was aligned with those of *S. cerevisiae SYN8* and *C. albicans SYN8*, and the BLAST results showed that *C. glabrata* Syn8p shares 40% identity with *S. cerevisiae* Syn8p and 33% identity with *C. albicans* Syn8p.

**FIGURE 2:**
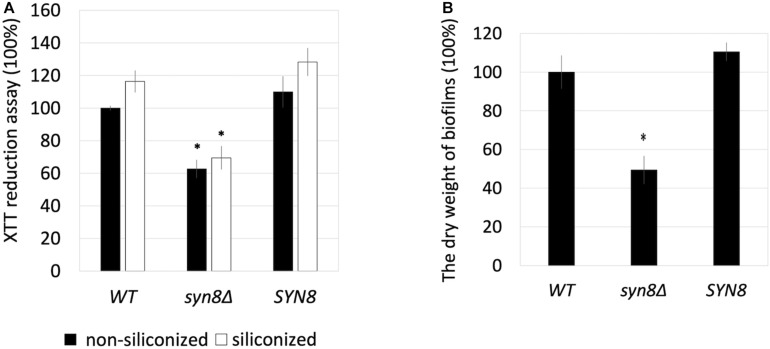
Effect of siliconized plates on biofilm formation. **(A)** The metabolic activities of biofilms of the wild type strain, *syn8*Δ mutant, and *SYN8* reintegrated strain were detected in non-siliconized plates and siliconized plates, respectively. **(B)** Biofilms of the wild type strain, *syn8*Δ mutant, and *SYN8* reintegrated strain were respectively formed in 12-well plates, and the dry weight was measured. The reported values are the means ± *SD* of three independent experiments. ^∗^*p* < 0.05 with Student’s *t*-test. ^∗^ vs. *WT* or *SYN8*.

Next, the growth rate of the *C. glabrata syn8*Δ strain was measured and compared with those of the wild type and a *SYN8* reintegrated strain. The *syn8*Δ mutant grew similarly to the wild type and reintegrated strains with a 24 h incubation in CSM medium, which was also used in the biofilm formation experiments (Supplementary Figure 3). After that, the biofilm formation of the *syn8*Δ mutant was compared to those of the wild type strain and the reintegration *SYN8* strain. On the non-siliconized plates made from polystyrene, the metabolic activity of the *syn8*Δ mutant cells was significantly lower than those of the wild type strain and the *SYN8* reintegrated strain when measured by XTT assay. In the siliconized plates (silicone coated polystyrene), the biofilm formation of the *syn8*Δ mutant showed almost the same proportional reduction as that on the non-siliconized plates, while the reintegration *SYN8* strain kept the same levels of biofilm formation compared to the wild type strain (Figure 2A). From these results, we used the non-siliconized plates in further experiments. To further compare the quantities of mature biofilm cells among the three strains, the biomass of the biofilms was measured. The dry weights of biofilms of the *syn8*Δ mutant were almost half as much as those of the wild type strain, while the reintegration *SYN8* strain showed no decrease in biofilm biomass (Figure 2B).

**FIGURE 3:**
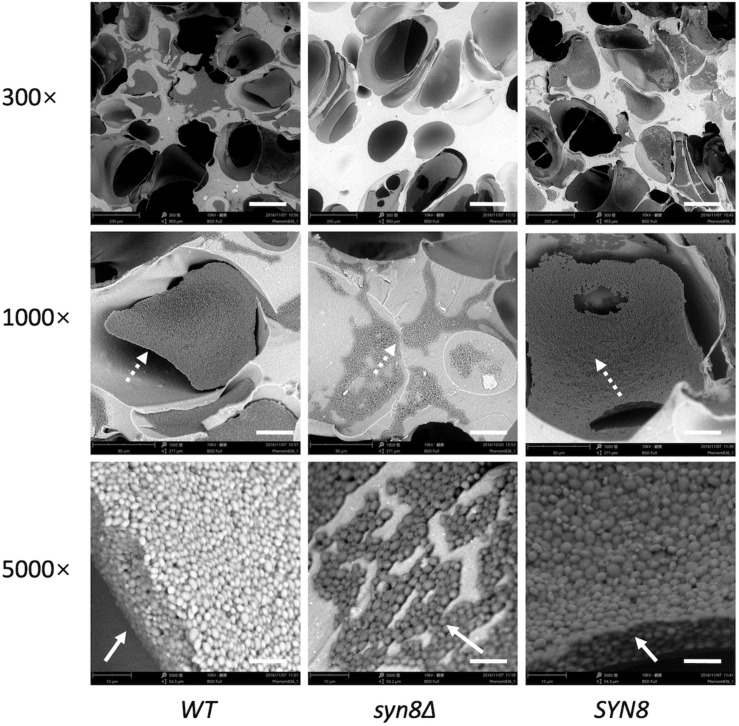
Morphology of 24 h biofilms. Biofilms of the wild type strain, *syn8*Δ mutant, and *SYN8* reintegrated strain were formed on silicone sponges in CSM medium for 24 h. Biofilms of these three strains were visualized by SEM at 300×, 1,000×, and 5,000× magnification. Scale bars indicate 150 μm in 300× images, 50 μm in 1,000× images and 10 μm in 5,000× images.

The biofilm structures of these three strains on silicone sponges were observed by SEM. The SEM images showed that biofilms of the wild type strain extend widely with many cells, while few cells aggregated in the biofilm of the *syn8*Δ mutant (Figure 3). The reintegration *SYN8* strain recovered this phenotype of the mutant strain, indicating that the *SYN8* gene is involved in the development of biofilms in *C. glabrata*. The reductions in metabolic activity, biomass, and biofilm development in the *syn8*Δ mutant suggested that the *SYN8* gene plays an important role in biofilm formation of *C. glabrata*.

Matrix analysis was performed to detect the carbohydrate and protein components in the matrix of biofilms. Table 4 shows the yield of total carbohydrate and protein separated from biofilm matrix formed by the wild type, the *syn8*Δ mutant, and the reintegration strain. The total carbohydrate and protein in the biofilm matrix formed by the *syn8*Δ mutant was much less than in the wild type and reintegration strains. However, when the amounts of carbohydrate and protein were normalized for the dry weight of the biofilm cells, the values showed no significant difference. This suggested that deletion of *SYN8* had no effect on the proportion of biofilm matrix components in *C. glabrata.*

**TABLE 4:** Biofilm matrix analysis for carbohydrate and protein components.

Strain	Carbohydrate (μg/mL)	Protein (μg/mL)	Carbohydrate/biomass (μg/mg)	Protein/biomass (μg/mg)
*WT*	309.18 ± 12.34	3.36 ± 0.49	240.28 ± 23.36	2.66 ± 0.74
*syn8*Δ	155.22 ± 8.36	1.72 ± 0.32	243.36 ± 7.76	2.725 ± 0.74
*SYN8*	408.49 ± 57.15	4.34 ± 0.21	245.19 ± 17.26	2.65 ± 0.69

*The reported values are the means ± SD of three independent experiments.*

**FIGURE 4:**
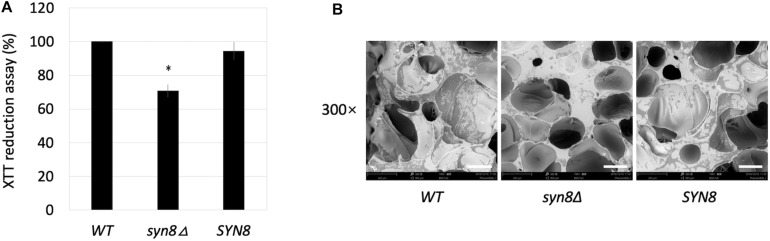
Adhesion ability. **(A)** The metabolic activities of biofilms (1.5 h) of the wild type, *syn8*Δ mutant, and *SYN8* reintegrated strains were tested by XTT reduction assay. The reported values are the means ± *SD* of three independent experiments. ^∗^*p* < 0.05 with Student’s *t*-test, ^∗^ vs. *WT* or *SYN8*. **(B)** The morphology of adhesion cells of the wild type, *syn8*Δ mutant, and *SYN8* reintegrated strains were visualized by SEM at 300 × magnification. Scale bars indicate 150 μm in 300× images.

The first step in biofilm formation is cell adherence to the surface. The adherent activity of the *syn8*Δ mutant during biofilm formation was also analyzed. After an initial 1.5 h of biofilm formation in siliconized plates, the non-adherent cells were washed away by PBS, and the metabolic activities of cells adherent to the surface of the plate bottom were measured by XTT assay. The *syn8*Δ mutant showed around 70% metabolic activity compared with the wild type and reintegration strains (Figure 4A). When observing the adhesion step by SEM imaging, the results also showed that there were far fewer cells of the *syn8*Δ mutant attached on the surface of the silicone compared with the wild type and reintegration strains (Figure 4B). Therefore, normal adhesion of biofilms seems to require Syn8p.

**FIGURE 5:**
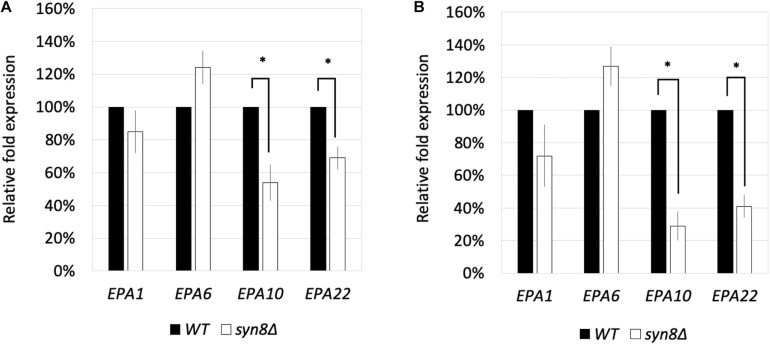
The transcriptional expression levels of *EPA1*, *EPA6*, *EPA10*, and *EPA22* in *C. glabrata* under planktonic cell growth conditions **(A)** and biofilm formation conditions **(B)**. The gene expression values were normalized by comparison to the expression of a housekeeping gene, *CgACT1*. 100% to the control group of wild type strain. Reported values indicate the means ± *SD* of at least three independent experiments. ^∗^*p* < 0.05 with Student’s *t*-test.

Because the *syn8*Δ mutant was observed to show poor adhesion, four adhesin genes, *EPA1*, *EPA6*, *EPA10*, and *EPA22*, were selected to investigate their transcriptional expression levels in the *syn8*Δ mutant and the control strains under planktonic cell growth and biofilm formation conditions. There were no significant differences in the transcriptional expression levels of *EPA1* and *EPA6* genes under either condition. However, *EPA10* and *EPA22* showed decreased expression in the *syn8*Δ mutant under both conditions compared with the wild type control (Figures 5A,B).

**FIGURE 6:**
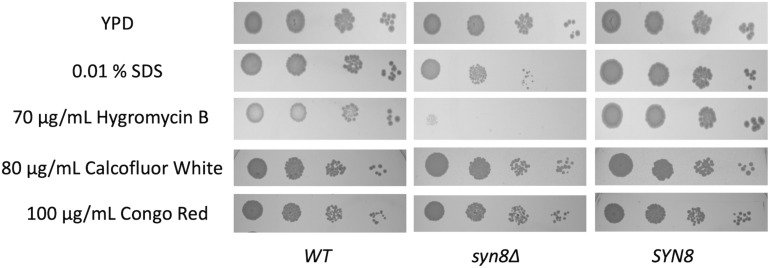
Susceptibility to antifungals by spot-dilution assays. Overnight cultures were diluted in 10-fold serial dilutions and spotted onto YPD plates containing 0.01% SDS, 70 μg/mL hygromycin B, 80 μg/mL calcofluor white, and 100 μg/mL Congo red, respectively.

Although we found that the *SYN8* gene contributes to biofilm formation and surface adhesion, it remained unclear whether this gene was related to stress resistance in biofilms of *C. glabrata*. Therefore, the growth of the *syn8*Δ mutant in the presence of several antimicrobial reagents was detected by spot dilution assay. Compared to the wild type and *SYN8* reintegrated strains, the *syn8*Δ mutant showed significant defects in growth with SDS and hygromycin B. However, deletion of *SYN8* seemed to show no change in susceptibility to calcofluor white and Congo red, which interfere with cell wall assembly (Figure 6).

Since these antimicrobial treatments show poor activity against biofilm cells when compared with planktonic cells, the MIC of hygromycin B to planktonic cells and the SMIC of hygromycin B to biofilm cells were measured. The MIC_50_ and MIC_90_ of planktonic cells of the *syn8*Δ mutant were respectively 7.8125 and 62.5 μg/mL for hygromycin B, values that were much lower than those of the wild type strain (62.5 and 250 μg/mL) and *SYN8* reintegrated strain (125 and 250 μg/mL). The SMIC_50_ and SMIC_90_ of the *syn8*Δ mutant biofilm cells were 125 and 1,000 μg/mL, respectively, which were much higher than those of its own planktonic cells. However, the SMIC_50_ of the wild type strain and the integrated strain were 500 and 1,000 μg/mL, respectively, and the SMIC_90_ of these strains was above 1,000 μg/mL (Table 5).

**TABLE 5:** MIC of planktonic cells and SMIC of biofilm cells to hygromycin B.

Strains	Planktonic cells		Biofilm cells	
	MIC_50_ (μg/mL)	MIC_90_ (μg/mL)	SMIC_50_ (μg/mL)	SMIC_90_ (μg/mL)
*WT*	62.5	250	500	>1,000
*syn8*Δ	7.8125	62.5	125	1,000
*SYN8*	125	250	1,000	>1,000

**FIGURE 7:**
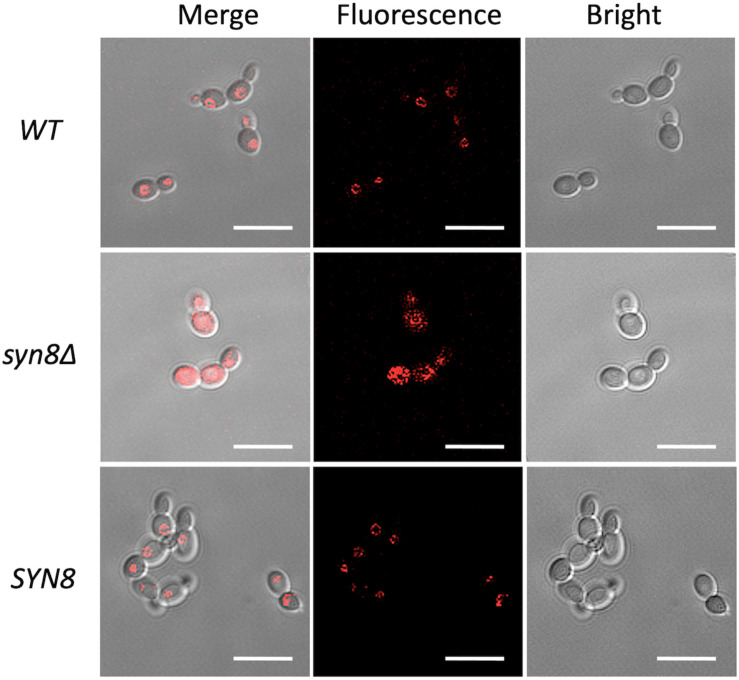
Characterization of the vacuolar morphology of *C. glabrata* strains. Living cells at exponential phase were stained by FM4-64 and visualized by bright-field and epifluorescence microscopy. Scale bars indicate 10 μm.

To synthesize mature proteins in cells normally, vacuolar functions such as vacuolar transport are necessary, and Syn8p is thought to be related to vacuolar function. To check the vacuolar function of the *syn8*Δ mutant, it was assayed for growth under various types of ionic stress. The *syn8*Δ mutant showed higher sensitivities to ZnCl_2_, MnCl_2_, and NaCl than the wild type strain (Supplementary Figure 4). In order to examine vacuole morphology, the fluorescent dye FM4-64 was used to stain vacuolar membranes and was viewed by CLSM. FM4-64 is a lipophilic styryl dye. Both the wild type and *SYN8* reintegrated strains showed clear circle vacuolar membranes that were stained by FM4-64. But *syn8*Δ mutant cells did not have a clear vacuole shape and looked to have large numbers of fragmented vacuoles (Figure 7). This result suggested that deletion of the *C. glabrata SYN8* gene resulted in defective vacuole morphology.

## Discussion

Syn8 protein in *S. cerevisiae* has been identified as the SNARE protein syntaxin 8, which plays a role in transport between the Golgi and prevacuolar compartments (PVC) (Lewis and Pelham, 2002). Previous findings have indicated that Syn8p may be a constituent of a SNARE complex with Vti1p, Pep12p, and Ykt6/Nyv1 proteins (Fischer Von Mollard and Stevens, 1999; Kweon et al., 2003).

Some studies on the relationship between biofilm formation and SNARE proteins in *C. albicans* and *S. cerevisiae* have been previously conducted. Pep12, which was mentioned above, has been reported to act as a t-snare protein and is classified into the Qa-/Syntaxin family (Lewis and Pelham, 2002). It has been reported to be involved in vacuolar transport and endocytosis in both *S. cerevisiae* and *C. albicans* (Kweon et al., 2003; Palanisamy et al., 2010). During biofilm formation of the *C. albicans pep12Δ* null mutant, there are notable defects in biofilm mass and biofilm integrity (Palanisamy et al., 2010). Another v-snare protein, Btn2, has also been demonstrated to play a role in biofilm formation in *S. cerevisiae* flor strains. Btn2p facilitates the trafficking of specific proteins from the late endosome to the Golgi apparatus. Furthermore, the deletion of *BTN2* affects biofilm formation due to alterations of the transcriptional level of the *FLO11* gene, a critical gene for biofilm formation in *S. cerevisiae* (Espinazo-Romeu et al., 2008). Our study is the first to demonstrate a role for a SNARE protein in biofilm formation of *C. glabrata.*

*Candida* biofilms have been reported to exist frequently in the bodies of patients with silicone implant devices and are highly resistant to antifungal drugs (Pristov and Ghannoum, 2019). Medical devices made of silicone materials include urinary catheters, ventriculoperitoneal shunts, and voice prosthesis (Kojic and Darouiche, 2004). Therefore, not only commercial microtiter plates but also siliconized plates were used for biofilm formation of *C. glabrata* in this study. The *syn8*Δ mutant decreased the metabolic activity of biofilms in both plate types. Furthermore, the *syn8*Δ mutant seemed to be unable to form layers of dense biofilm structures and construct the same biofilm biomass as the wild type. Our *in vitro* model results strongly suggest that *SYN8* is required for proper *C. glabrata* biofilm formation. Further work will be need to elucidate the function of this gene in biofilm model *in vivo*.

Mature biofilm structures consist of yeast cell layers and extracellular matrix, which mainly includes carbohydrates and proteins (Silva et al., 2009). The protein and carbohydrate amounts were also reduced in the *syn8*Δ mutant, although the absence of the *SYN8* gene did not influence the ratio of extracellular matrix components to biofilm biomass. Moreover, deletion of the *SYN8* gene in *C. glabrata* resulted in a decrease in the adhesion to the surface of the silicone plates. Since adhesion and production of extracellular matrix are significant steps to from biofilms of *C. glabrata*, these results suggested that *SYN8* might affect not only the adhesion ability in the early stage of biofilm formation, but also the extracellular matrix production in the late stage of biofilm formation.

The *EPA* gene family is a major group of adhesin-like glycosylphosphatidylinositol-cell-wall proteins (GPI-CWPs) of *C. glabrata*. Epa1p plays a critical role in adherence in *C. glabrata*. Epa6p has also been shown to play an important role during biofilm formation although it is not involved in adherence to the surface of plates (Iraqui et al., 2005). *EPA1*, *EPA6*, and *EPA10* share the same homolog in *S. cerevisiae*, *FLO1*, while the *EPA22* homolog in *S. cerevisiae* is *FLO10*. The Flo protein family in *S. cerevisiae* has been reported to mediate yeast flocculation, adherence, and biofilm formation (Verstrepen and Klis, 2006). In order to clarify the relationship between *EPA* gene expression and *SYN8* disruption in *C. glabrata*, the transcriptional expressions of *EPA1*, *EPA6, EPA10*, and *EPA22* during planktonic cell growth and biofilm formation were analyzed in the *syn8*Δ mutant. Since the expression levels of both *EPA10* and *EPA 22* in the *syn8*Δ mutant were much lower in biofilm formation than planktonic cell growth, these proteins may be needed for adhesion activity in biofilms of *C. glabrata.* Further research into these two *EPA* genes will be required to determine their relationship with the *SYN8* gene.

In addition, testing the resistance to several stressors demonstrated that the deletion of *SYN8* led to increased susceptibility to hygromycin B especially, not only in planktonic cells but also in biofilm cells. Hygromycin B inhibits translocation of mRNA and tRNA on the ribosome and interferes with decoding fidelity. It is widely used in veterinary medicine and in selection for cell culture (Borovinskaya et al., 2008). Based on previous research on *S. cerevisiae*, many mutants such as *VPS1*, *VPS34*, *VPS45*, and *VPS54* with vacuole trafficking or functional defects show hypersensitivity to hygromycin B (Banuelos et al., 2010; Smaczynska-de Rooij et al., 2010). Moreover, these *VPS* mutants have decreased growth in the presence of various ions, similar with the *syn8*Δ mutant in this study. This could be because vacuoles act as storage vessels for a wide variety of ions, responding to ionic shock and promoting intracellular ion homeostasis. For instance, Na^+^ and K^+^ are major cations in the cytosol, while they are minor components in the vacuolar pools (Rodríguez-Navarro, 2000). Excessive concentrations of Mg^2+^ and Zn^2+^ can become harmful to cells (Simm et al., 2007). In addition, our CLSM images showed that the vacuole morphologies of the *syn8*Δ mutant were abnormal when compared to the wild type strain. In *S. cerevisiae*, fragmented vacuoles are observed in the *VPS54* null mutant (Raymond et al., 1992). From our findings, the *syn8*Δ mutant of *C. glabrata* appeared to have defective vacuolar morphology and the detail study on the role of *SYN8* gene in the vacuolar function is expected in future research.

Vps proteins of *S. cerevisiae* have been reported to be required for the assembly of SNARE complexes during fusion between the trans-Golgi network and PVC. For example, some Vps proteins, together with the vacuolar SNARE proteins, function in the fusion of multiple transport intermediates with the vacuole (Sato et al., 2000). Some *C. albicans* Vps proteins, like Vps1p, have also been reported to be involved in biofilm formation (Bernardo et al., 2008). Therefore, it has been proposed that SNARE proteins and Vps proteins may act together on the secretory pathway, which plays a role in *Candida* virulence, such as biofilm formation (Rollenhagen et al., 2020).

## Data Availability Statement

The raw data supporting the conclusions of this article will be made available by the authors, without undue reservation.

## Author Contributions

XC and SK conceived the study and wrote the manuscript. XC performed the experiments. XC and SI collected and analyzed the data. TK and HC gave support. All authors contributed to the article and approved the submitted version.

## Conflict of Interest

The authors declare that the research was conducted in the absence of any commercial or financial relationships that could be construed as a potential conflict of interest.

## Supplementary Material

Supplementary Figure 1 Biofilm formation by 101 gene mutants of *C. glabrata* by XTT assay. 100% to the reference strain.

Supplementary Figure 2 The transcriptional expressions of three *C. glabrata* genes in mature (24 h) biofilm cells compared with planktonic cells.

Supplementary Figure 3 The growth rates of the wild type strain, *syn8*Δ mutant and *SYN8* reintegrated strain.

Supplementary Figure 4 Susceptibility to antifungals by spot-dilution assays. Overnight cultures were diluted in 10-fold serial dilutions and spotted onto YPD plates containing 4 mM ZnCl_2_, 10 mM MnCl_2_, 1 M KCl, and 1 M NaCl, respectively.

Supplementary Table 1 Biofilms of mutants in percentages relative to the reference strain (100%).
