# Supplementary figures and images for: The Lack of SNARE Protein Homolog Syn8 Influences Biofilm Formation of Candida glabrata
Source: Front Cell Dev Biol. 2021 Feb 12;9:607188. doi: 10.3389/fcell.2021.607188 (PMC7907433; doi:10.3389/fcell.2021.607188)

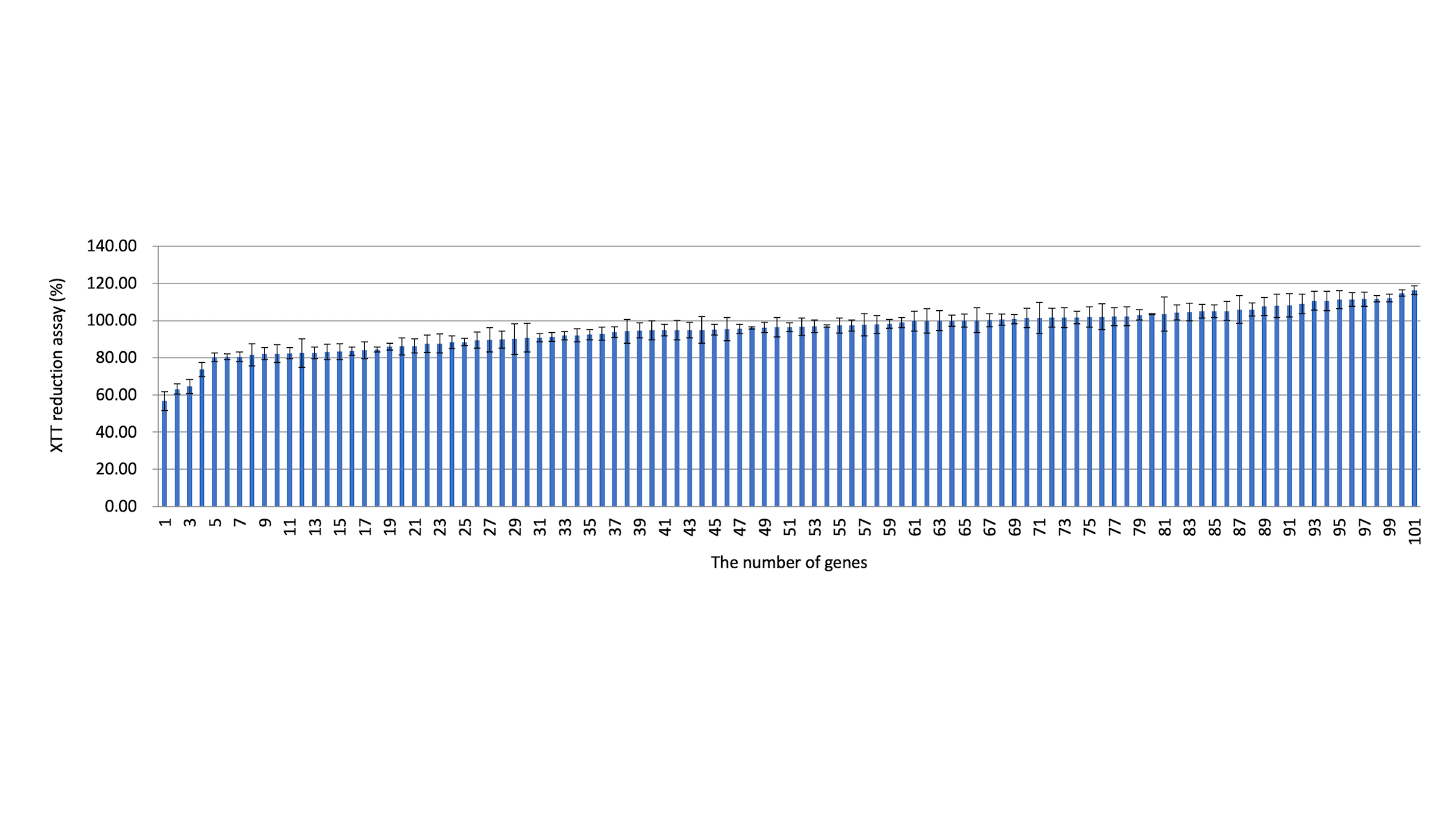

Supplement: Supplementary Figure 1 — Biofilm formation by 101 gene mutants of C. glabrata by XTT assay. 100% to the reference strain. [file Image_1.tiff]

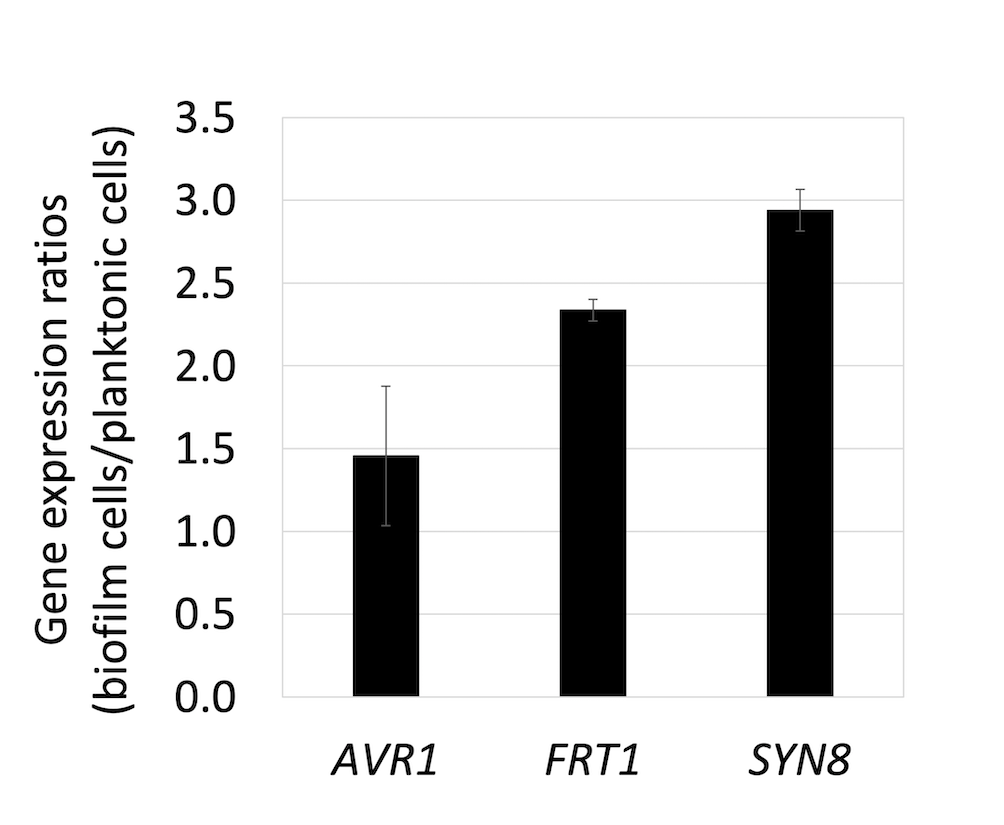

Supplement: Supplementary Figure 2 — The transcriptional expressions of three C. glabrata genes in mature (24 h) biofilm cells compared with planktonic cells. [file Image_2.TIFF]

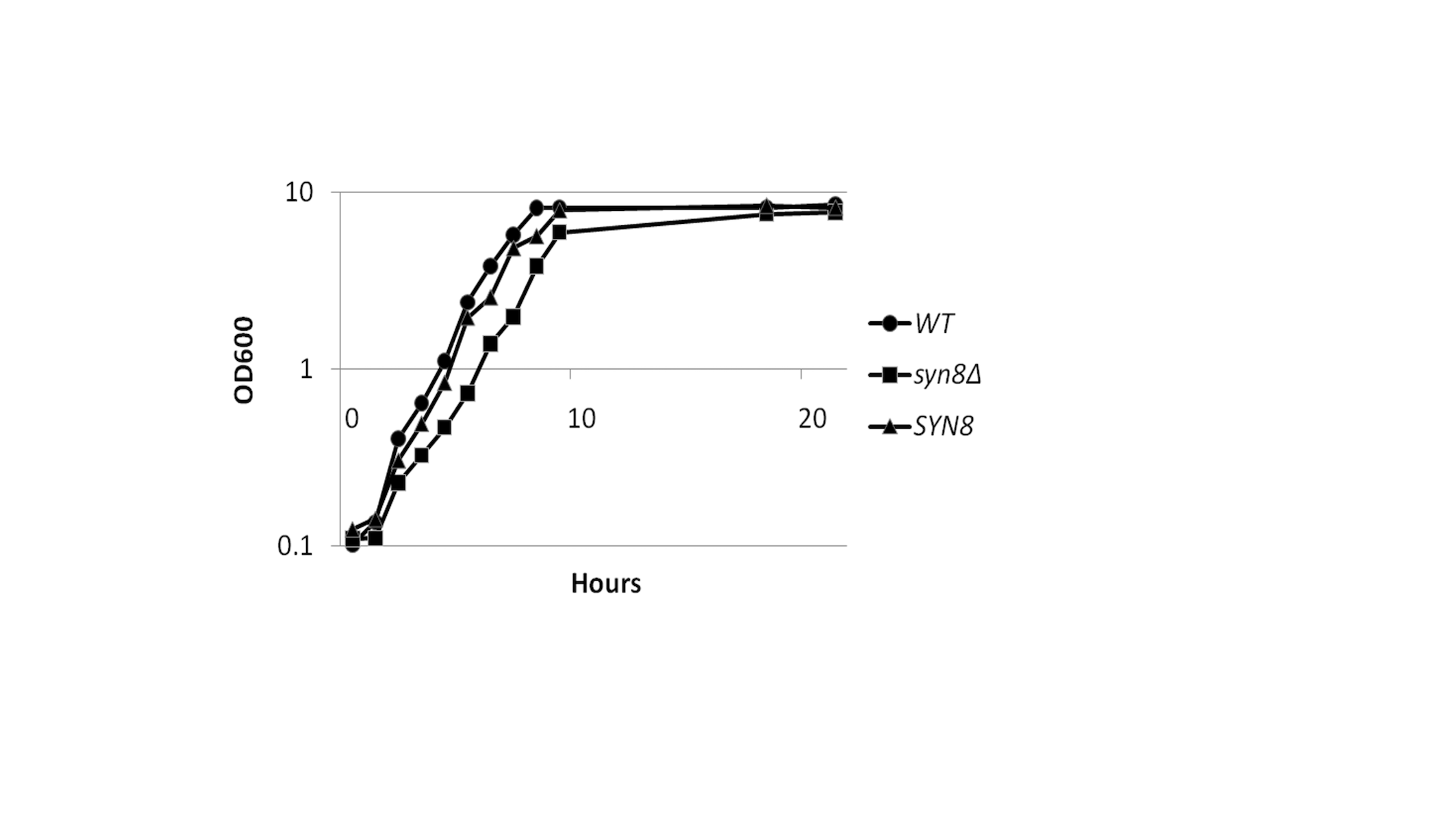

Supplement: Supplementary Figure 3 — The growth rates of the wild type strain, syn8Δ mutant and SYN8 reintegrated strain. [file Image_3.TIFF]

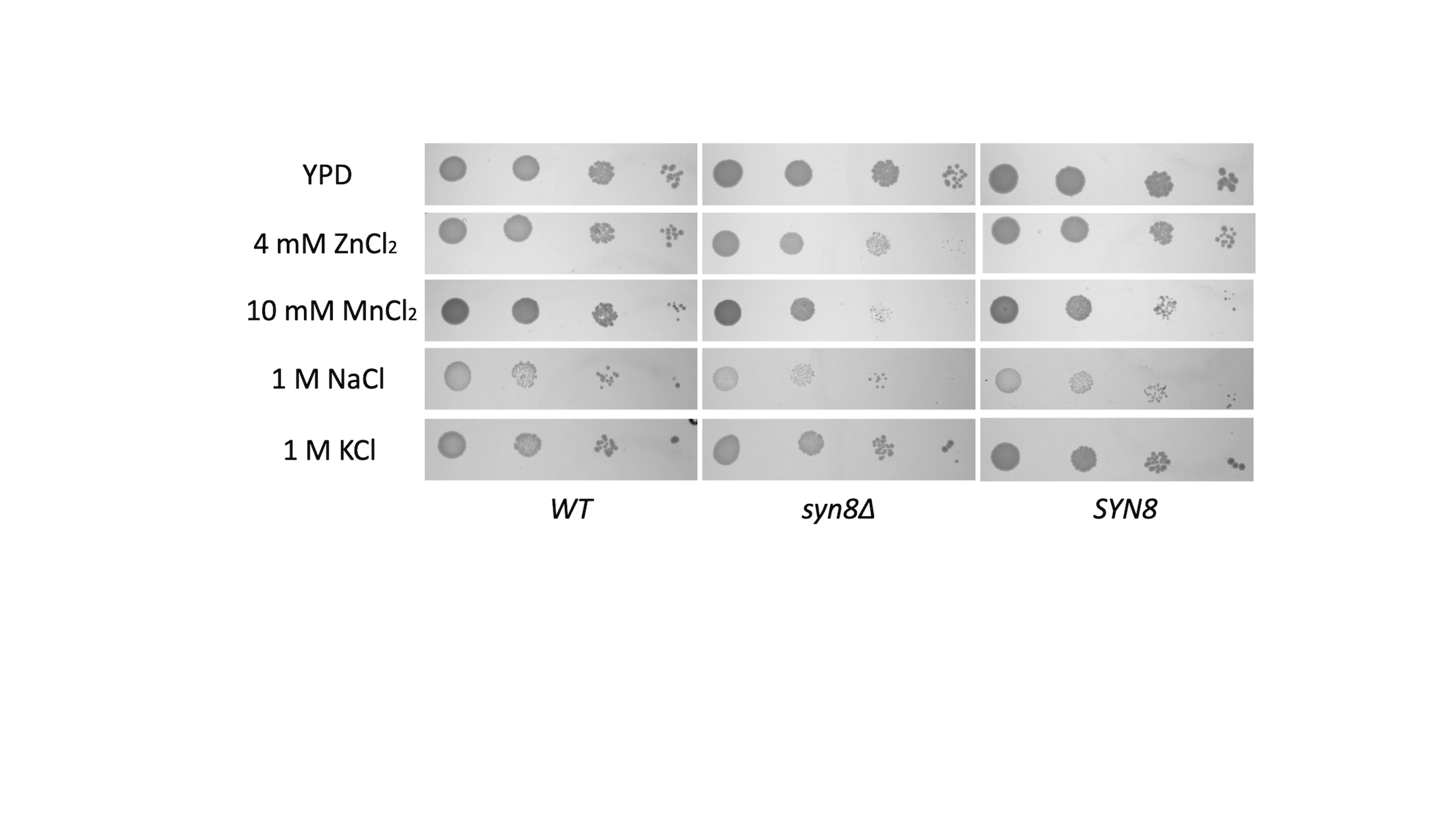

Supplement: Supplementary Figure 4 — Susceptibility to antifungals by spot-dilution assays. Overnight cultures were diluted in 10-fold serial dilutions and spotted onto YPD plates containing 4 mM ZnCl2, 10 mM MnCl2, 1 M KCl, and 1 M NaCl, respectively. [file Image_4.TIFF]
