# Supplementary material for: The Lack of SNARE Protein Homolog Syn8 Influences Biofilm Formation of Candida glabrata
Source: Front Cell Dev Biol. 2021 Feb 12;9:607188. doi: 10.3389/fcell.2021.607188 (PMC7907433; doi:10.3389/fcell.2021.607188)
Supplement: Supplementary Table 1 — Biofilms of mutants in percentages relative to the reference strain (100%). [file Table_1.pdf]

No.	Systematic names in *C. glabrata*	Biofilm (%)	STDEV	Ortholog genes/best hits in *S. cerevisiae*
1	CAGL0G08316g	56.75	5.12	ARV1
2	CAGL0H06325g	63.15	2.68	SYN8
3	CAGL0G06314g	64.53	3.87	FRT2
4	CAGL0G06358g	73.70	3.79	SNC1
5	CAGL0M10769g	80.31	2.23	ALG12
6	CAGL0E01727g	80.58	1.56	YPS1
7	CAGL0B00528g	80.62	2.57	LRE1
8	CAGL0J05159g	81.67	5.98	no similarity
9	CAGL0M05489g	82.25	3.30	KTR3
10	CAGL0M06985g	82.26	4.94	ERS1
11	CAGL0A02431g	82.51	2.98	YPS7
12	CAGL0J05236g	82.54	7.71	LAS21
13	CAGL0D04422g	82.68	3.05	YOR390W
14	CAGL0C04213g	83.16	4.23	RCR2
15	CAGL0F01287g	83.36	4.39	GAS5
16	CAGL0E05654g	83.59	2.33	PGC1
17	CAGL0K04939g	84.21	4.57	YNL217W
18	CAGL0B04763g	84.51	1.30	LDB16
19	CAGL0M12969g	86.05	1.83	YIL077C
20	CAGL0G08668g	86.22	4.61	SUN4 or SIM1
21	CAGL0L07854g	86.39	3.76	CWH43
22	CAGL0E06644g	87.50	4.69	FLO10
23	CAGL0E03113g	87.72	4.99	RSR1
24	CAGL0M09229g	88.39	3.32	YJR085C
25	CAGL0L05082g	88.53	1.97	YKL077W
26	CAGL0L07326g	89.52	4.35	DUN1
27	CAGL0M01826g	89.76	6.52	ECM33
28	CAGL0K02893g	89.84	4.49	OSH7
29	CAGL0H08129g	90.11	8.32	DFG16
30	CAGL0K03025g	90.80	7.65	TVP18
31	CAGL0E03542g	90.83	2.27	SPA2
32	CAGL0B04719g	91.28	2.46	YCL002C
33	CAGL0M05027g	91.95	2.15	BCH1

34	CAGL0L00157g	92.15	3.56	no similarity
35	CAGL0G10219g	92.54	2.75	FLO5
36	CAGL0K06897g	92.90	3.51	YBR225W
37	CAGL0D00286g	93.86	2.80	no similarity
38	CAGL0H09328g	94.27	6.31	QCR9
39	CAGL0F04851g	94.73	4.12	NCA2
40	CAGL0L00227g	94.84	5.04	no similarity
41	CAGL0C03179g	94.92	3.16	SEC22
42	CAGL0E06688g	95.00	5.26	FLO10
43	CAGL0E04686g	95.02	4.18	YOS9
44	CAGL0C05159g	95.03	7.22	MAM3
45	CAGL0L05626g	95.24	2.84	SPT10
46	CAGL0F02827g	95.32	6.26	DFM1
47	CAGL0G05918g	95.63	2.46	CHS7
48	CAGL0G00682g	96.10	0.76	ERP1
49	CAGL0I05098g	96.32	2.76	SBH1
50	CAGL0H03333g	96.48	5.15	KXD1
51	CAGL0L03828g	96.53	2.32	CYB5
52	CAGL0J01463g	96.69	4.65	CWP1/YKL096W
53	CAGL0C04939g	97.04	3.48	YJR107W
54	CAGL0G01188g	97.07	0.61	ORM1
55	CAGL0J09702g	97.41	4.02	ACK1
56	CAGL0A01584g	97.49	3.01	AGA2
57	CAGL0A00627g	97.74	5.97	ERP6
58	CAGL0D01232g	97.95	4.82	MRL1
59	CAGL0A03146g	98.29	2.43	YDR381C-A
60	CAGL0K05401g	98.98	2.76	ERV2
61	CAGL0H04191g	99.84	5.34	ATP18
62	CAGL0G08217g	99.91	6.52	ERF2
63	CAGL0K10120g	99.94	5.34	PET100
64	CAGL0L10142g	99.96	3.02	RSB1
65	CAGL0M13233g	100.03	3.55	MNR2
66	CAGL0F01639g	100.19	6.65	PER33
67	CAGL0H09482g	100.29	3.55	BUD25
68	CAGL0J11374g	100.60	2.99	no similarity

69	CAGL0H06732g	100.82	2.55	UBP7
70	CAGL0G09449g	101.40	5.19	CRH1
71	CAGL0K03685g	101.41	8.31	PKR1
72	CAGL0M06347g	101.58	4.97	YPC1
73	CAGL0M13255g	101.62	5.37	YET1
74	CAGL0L08602g	101.73	3.49	PPX1
75	CAGL0E00231g	102.01	5.51	no similarity
76	CAGL0J08756g	102.07	6.82	HRD3
77	CAGL0I03432g	102.20	4.81	MHF2
78	CAGL0L04642g	102.28	5.05	ALE1
79	CAGL0C01617g	103.02	2.75	no similarity
80	CAGL0J06490g	103.55	0.34	CUE1 or CUE4
81	CAGL0F07953g	103.55	9.22	SPG1
82	CAGL0D02134g	104.42	4.07	YKL133C or MGR3
83	CAGL0F07931g	104.57	4.58	MIC26
84	CAGL0G07447g	105.15	3.62	YPL168W
85	CAGL0I06644g	105.18	3.42	SED1
86	CAGL0J01699g	105.23	5.12	YPR010C-A
87	CAGL0B00594g	105.99	7.35	AGE1
88	CAGL0G05522g	106.00	3.51	no similarity
89	CAGL0M05929g	107.59	4.81	PAM17
90	CAGL0D00704g	107.96	6.28	YET3
91	CAGL0H02563g	108.19	6.31	AGE1
92	CAGL0C04829g	109.00	5.31	NNF1
93	CAGL0C03575g	110.60	5.09	DAN4
94	CAGL0K00110g	110.64	5.16	HPF1
95	CAGL0G07040g	111.25	4.89	OST6
96	CAGL0K06127g	111.30	3.64	ECM11
97	CAGL0B04389g	111.55	3.81	CHS3
98	CAGL0E01045g	111.75	1.62	NBP2
99	CAGL0J04092g	112.18	1.99	YOR223W
100	CAGL0I06270g	114.88	1.76	QCR8
101	CAGL0M00924g	116.31	2.32	ECM30
